# Performance of Metagenomic Next-Generation Sequencing for the Diagnosis of Viral Meningoencephalitis in a Resource-Limited Setting

**DOI:** 10.1093/ofid/ofaa046

**Published:** 2020-02-08

**Authors:** Nguyen Thi Thu Hong, Nguyen To Anh, Nguyen Thi Hoang Mai, Ho Dang Trung Nghia, Le Nguyen Truc Nhu, Tran Tan Thanh, Nguyen Hoan Phu, Xutao Deng, H Rogier van Doorn, Nguyen Van Vinh Chau, Eric Delwart, Guy Thwaites, Le Van Tan

**Affiliations:** 1 Oxford University Clinical Research Unit, Ho Chi Minh City, Vietnam; 2 Pham Ngoc Thach University of Medicine, Ho Chi Minh City, Vietnam; 3 Department of Medicine, Vietnam National University, Ho Chi Minh City, Vietnam; 4 Vitalant Research Institute, San Francisco, California, USA; 5 Department of Laboratory Medicine, University of California, San Francisco, California, USA; 6 Centre for Tropical Medicine and Global Health, Nuffield Department of Medicine, University of Oxford, Oxford, UK; 7 Hospital for Tropical Diseases, Ho Chi Minh City, Vietnam

**Keywords:** metagenomics, next-generation sequencing, nanopore, MinION, meningoencephalitis

## Abstract

**Background:**

Meningoencephalitis is a devastating disease worldwide. Current diagnosis fails to establish the cause in ≥50% of patients. Metagenomic next-generation sequencing (mNGS) has emerged as pan-pathogen assays for infectious diseases diagnosis, but few studies have been conducted in resource-limited settings.

**Methods:**

We assessed the performance of mNGS in the cerebrospinal fluid (CSF) of 66 consecutively treated adults with meningoencephalitis in a tertiary referral hospital for infectious diseases in Vietnam, a resource-limited setting. All mNGS results were confirmed by viral-specific polymerase chain reaction (PCR). As a complementary analysis, 6 viral PCR-positive samples were analyzed using MinION-based metagenomics.

**Results:**

Routine diagnosis could identify a virus in 15 (22.7%) patients, including herpes simplex virus (HSV; n = 7) and varicella zoster virus (VZV; n = 1) by PCR, and mumps virus (n = 4), dengue virus (DENV; n = 2), and Japanese encephalitis virus (JEV; n = 1) by serological diagnosis. mNGS detected HSV, VZV, and mumps virus in 5/7, 1/1, and 1/4 of the CSF positive by routine assays, respectively, but it detected DENV and JEV in none of the positive CSF. Additionally, mNGS detected enteroviruses in 7 patients of unknown cause. Metagenomic MinION-Nanopore sequencing could detect a virus in 5/6 PCR-positive CSF samples, including HSV in 1 CSF sample that was negative by mNGS, suggesting that the sensitivity of MinION is comparable with that of mNGS/PCR.

**Conclusions:**

In a single assay, metagenomics could accurately detect a wide spectrum of neurotropic viruses in the CSF of meningoencephalitis patients. Further studies are needed to determine the value that real-time sequencing may contribute to the diagnosis and management of meningoencephalitis patients, especially in resource-limited settings where pathogen-specific assays are limited in number.

Meningoencephalitis is a devastating clinical condition worldwide, but especially in tropical and resource-limited settings [1]. Although viruses are regarded as the most common causes of meningoencephalitis, the viruses responsible vary between geographic locations and are influenced by the emergence of pathogens such as Nipah virus, enterovirus A71, and Zika virus [2–4]. However, detecting many of these viruses is challenging, especially when most conventional diagnostic tests are pathogen specific (eg, polymerase chain reaction [PCR] for herpes simplex virus) and limited in number, especially in resource-limited settings. Even in well-equipped laboratories, a causative virus has only been established in <60% of patients [5–8].

Over the last decade, advanced sequencing technologies have emerged as a single pan-pathogen assay for the sensitive detection of known and unknown microorganisms, especially viruses, in cerebrospinal fluid (CSF) [6, 9, 10]. As part of our pathogen discovery, using a viral metagenomics-based approach, we previously identified a novel cyclovirus (CyCV-VN) in 4% of Vietnamese patients presenting with meningoencephaitis of unknown cause [11], although the pathogenic relevance of this novel circovirus species remains uncertain. From a diagnostic perspective, a recent prospective study in the United States compared the diagnostic performance of routine diagnostic tests with metagenomic next-generation sequencing (mNGS) and showed that mNGS detected a bacteria or virus in the CSF of 13 of 58 patients presenting with meningoencephalitis who were negative for or not assessed with routine diagnostic tests [6]. Otherwise, studies to date have been either case reports or retrospectively performed with small sample sizes [12], but few have been carried out in resource-limited settings like Vietnam. Such studies would have significant implications for both disease surveillance and patient management. Herein, we report the results of a study assessing the potential of metagenomics to detect a broad range of viruses in the CSF of consecutively treated adults with meningoencephalitis presenting to a tertiary referral hospital in southern Vietnam.

## METHODS

### Setting, Patient Enrollment, and Data Collection

The present study was conducted in a brain infection ward of the Hospital for Tropical Diseases (HTD) in Ho Chi Minh City, Vietnam, between January 2015 and September 2016. HTD is a tertiary referral hospital for patients, especially adults, with infectious diseases, including encephalitis, from the southern provinces of Vietnam with a population of >40 million.

One of the aims of the study was to improve diagnosis in patients with meningoencephalitis using metagenomic next-generation sequencing. We enrolled consecutive adult patients (≥18 years) with an indication for lumbar puncture admitted to the study site during the study period. Patients were excluded if pyogenic bacterial meningitis (cloudy or pus-like CSF) was suspected, lumbar puncture was contra-indicated, or no written informed consent was obtained was obtained from the patient or their relatives.

As per the study protocol, CSF samples were collected, alongside demographic and clinical data (including discharge outcome) and the results of routine diagnostic testing. After collection, all clinical specimens were stored at –80°C for subsequent analyses, including assessment of mNGS performance against that of routine diagnostic assays. Here we focused our analysis on patients with meningoencephalitis regardless of the results of routine diagnosis. Additionally, as negative controls, 1 CSF from a patient presenting with cerebral hemorrhage and 1 from a patient with laboratory-confirmed anti-N-methyl-D-aspartate receptor [13] were also included.

### Routine Diagnosis

As part of routine care at HTD, CSF specimens of patients presenting with brain infections were cultured and/or examined by microscopy for detection of bacterial/fungal/*Mycobacterium tuberculosis* infection with the use of standard methods when appropriate (Supplementary Table 1). Herpes simplex virus (HSV) PCR was carried out in patients presenting with clinically suspected meningoencephalitis. Varicella zoster virus (VZV) PCR, serological testing for IgM against dengue virus (DENV), Japanese encephalitis virus (JEV), or MuV was performed if clinically indicated and testing for other pathogens (HSV) was negative [8].

### Illumina MiSeq/MinION–Based Viral Metagenomics

#### Sample Pretreatments and Nucleic Acid Isolation

**Figure 1.**
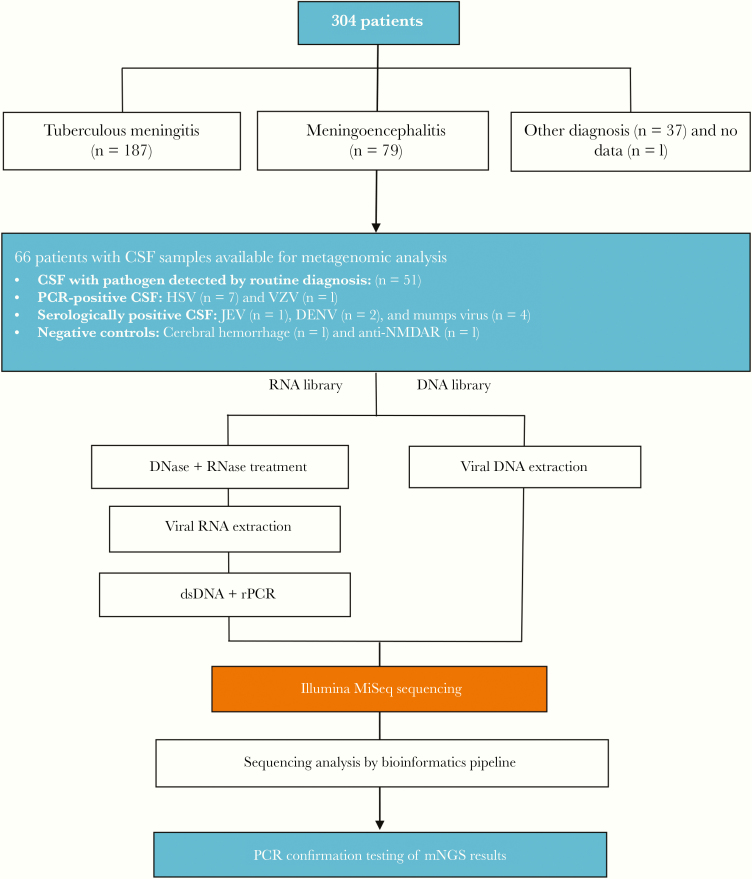
Flowchart illustrating an overview about the DNA and RNA virus workflows. Abbreviations: CSF, cerebrospinal fluid; DENV, dengue virus; ds, double-stranded; JEV, Japanese encephalitis virus; mNGS, metagenomic next-generation sequencing; PCR, polymerase chain reaction.

To allow for the detection of both RNA and DNA viruses, each CSF sample was subjected to 2 different metagenomic approaches, namely RNA virus and viral DNA virus workflows (Figure 1). For the former, 200 µL of CSF was first pretreated with 2 U/µL of turbo DNase (Ambion, Life Technology, Carlsbad, CA, USA) and 0.4 U/µL RNase 1 (Ambion) at 37°C for 30 minutes by DNase and RNase, followed by nucleic acid (NA) isolation using the QIAamp viral RNA kit (QIAgen GmbH, Hilden, Germany). For the latter, viral DNA was directly isolated from 200 µL of CSF samples without the nuclease treatment step using the DNeasy blood and tissue kit (QIAgen GmbH). Finally, viral RNA/DNA of both workflows was recovered in 50 µL of elution buffer.

#### Double-Stranded DNA Synthesis and Random Amplification of Extracted Viral RNA

Double-stranded DNA was synthesized from isolated viral RNA using a set of 96 nonribosomal random primer, followed by PCR amplification to enrich for viral RNA before sequencing as previously described [14–16]. In brief, 10 µL of extracted viral RNA was converted into double-stranded DNA (dsDNA) using FR26RV-Endoh primers [16], Super Script III enzyme (Invitrogen, Carlsbad, CA, USA), RNase OUT (Invitrogen), exo-Klenow fragment (Ambion), and Ribonuclease H (Ambion). Subsequently, the synthesized dsDNA was randomly amplified using FR20RV primer (5’-GCCGGAGCTCTGCAGATATC-3’). The obtained random PCR product was then purified with use of Agencourt AMPure XP beads (Beckman coulter) and quantified using the Qubit dsDNA HS kit (Invitrogen).

#### Next-Generation Sequencing

One ng of the purified random PCR product of the RNA virus workflow and isolated viral DNA of the DNA virus workflow was subjected to the library preparation step using the Nextera XT sample preparation kit (Illumina, San Diego, CA, USA), following the manufacturer’s instructions. Samples were multiplexed using the combinatorial indexing strategy (ie, only 1 index might be shared between samples). The resulting libraries of both workflows were separately sequenced using MiSeq reagent kits, version 3 (600 cycles; Illumina), in a MiSeq platform (Illumina), following the manufacturer’s instructions. All the experiments were performed in molecular diagnostic facilities that consist of 3 physically separated laboratories for reagent preparation, extraction, and library preparation and sequencing. These were used a unidirectional workflow.

#### MinION Library Preparation and Sequencing

A subset of 6 CSF samples in which a virus was detected by PCR and/or mNGS was selected for a complementary analysis using MinION sequencer (Oxford Nanopore Technologies). MinION libraries were prepared using either extracted DNA or random amplified products synthesized as described above using the 1D Native Barcoding Genomic DNA kit (ONT, Oxford, UK), following the manufacturer’s protocol. The 6 CSF samples and a nontemplate control (each was assigned to unique barcodes) were sequenced in 1 single run using R9.4 flow cells (ONT). Base-calling of MinION reads was performed using MinKNOW (ONT), followed by demultiplexing of the obtained reads using Porechop (https://github.com/rrwick/Porechop).

#### Sequence Analysis of the Obtained Metagenomic Reads

The mNGS data generated by the Illumina MiSeq platform were analyzed using an in-house viral metagenomic pipeline running on a 36-node Linux cluster available through Vitalant Research Institute, San Francisco, to identify the presence of viral sequences in the tested specimens, as previously described [17, 18]. In brief, after filtering out duplicate reads and reads belonging to human and bacterial genomes, and with adaptors and low-quality reads trimmed, the remaining reads were de novo assembled. The resulting contigs and singlet reads were then aligned against a customized viral proteome database extracted from the NCBI’s RefSeq and NR databases using a Basic Local Alignment Search Tool (BLAST)–based approach. Next, the candidate viral reads were aligned against a nonredundant nonvirus protein database to remove any false-positive reads (ie, reads with expected [E] values higher than those in viral protein databases) using DIAMOND [19]. Any viral-like sequence with an E value of ≤10^–5^ was considered a significant hit and was then manually checked by BLASTX to further exclude false-positive hits. Finally, a reference-based mapping approach was employed to assess the level of identity and genome coverage of the corresponding viruses.

Analysis of MinION reads was carried out using Taxonomer [20], a publicly available metagenomics pipeline, which incorporates an interactive results visualization function.

### PCR Confirmation of Viral Hits Detected by Metagenomics and Expanded PCR Testing

Because of the uncertainty in the diagnostic performance of mNGS and the focus of the present study, we performed specific PCRs to confirm mNGS hits matched with the genomes of neurotropic viruses. The PCR experiments were either carried out on leftover extracted RNA/DNA after the mNGS library preparation experiments or on newly extracted nucleic acids (NA). An mNGS result was only considered positive if it was subsequently confirmed by a corresponding viral PCR analysis of the original NA materials derived from corresponding individual samples. All PCR primers and probes used were derived from previous publications [21–23], including a real-time reverse transcription PCR (RT-PCR) for generic detection of enteroviruses.

Because of the focus of the present study, viruses of unknown neurotropic property and well-known contaminants of the mNGS data set were not pursued further by subsequent PCR analysis.

Unless specified above, all the laboratory experiments and bioinformatics analyses were carried out at the Oxford University Clinical Research Unit in Ho Chi Minh City, Vietnam.

### GenBank Accession Numbers

Metagenomics data were deposited at NCBI (GenBank) under SRA accession number PRJNA58865.

### Ethics

This clinical study received approvals from the Institutional Review Board of the HTD and the Oxford Tropical Research Ethics Committee of the University of Oxford. Written informed consent was obtained from each study participant or relative (if the patient was unconscious).

## RESULTS

### Baseline Characteristics of the Patients Included for mNGS

During the study period, a total of 304 patients were enrolled in the clinical study, including patients with tuberculous meningitis (n = 187), meningoencephalitis (n = 79), another berculous meningitis diagnostic arm have been published elsewhere [24]. Of the 79 patients with a discharge diagnosis of meningoencephalitis, 66 (84%) had CSF samples available for metagenomic analysis (Figure 1). These patients were the focus of the present study regardless of the results of routine diagnosis.

**Figure 2.**
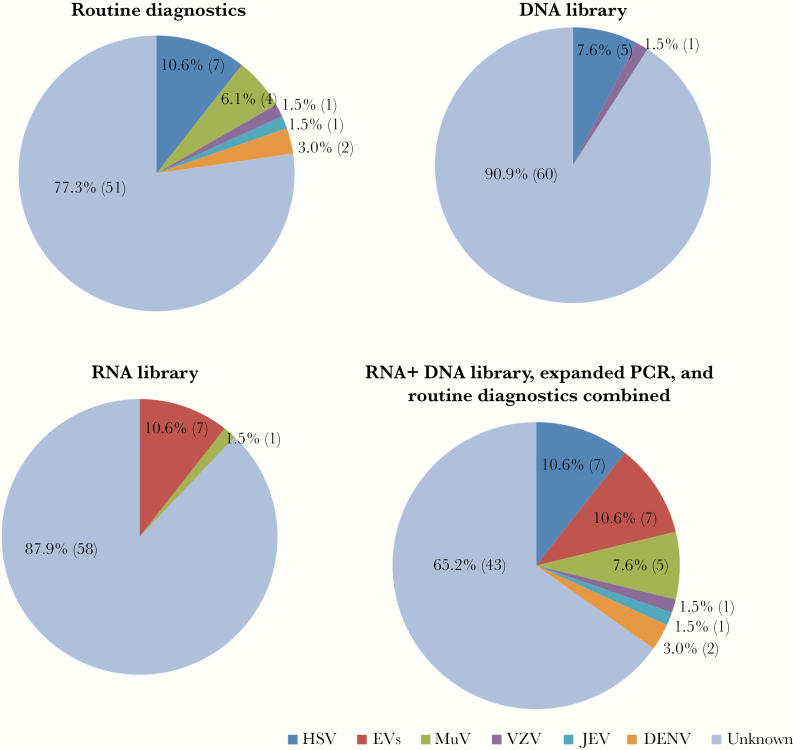
Results of metagenomic investigations using DNA/RNA workflows and routine diagnostics as well as expanded polymerase chain reaction testing. Abbreviations: DENV, dengue virus; EV, enterovirus; HSV, herpes simplex virus; JEV, Japanese encephalitis virus; MuV, mumps virus; PCR, polymerase chain reaction; VZV, varicella zoster virus.

The baseline characteristics of the 66 patients included in the study are presented in Table 1. HIV testing was carried out in 24 patients; none were positive. Males were predominant. On admission, 35% of the patients were comatose (Glasgow Coma Score < 13). Routine diagnostic tests identified a virus in 15/66 (22.7%) patients (Figure 2; Supplementary Table 2), with HSV being the most common cause (n = 7), followed by MuV (n = 4), DENV (n = 2), JEV (n = 1), and VZV (n = 1) (Figure 2). One patient died, and almost all (n = 58) had some neurological deficit at discharge from the hospital (Table 1).

**Table 1.** Baseline Characteristics of the Study Patients and Patients Infected With HSV/EVs/Mumps Virus

	Total (n = 66)^a^	HSV (n = 7)^b^	EVs (n = 7)^c^	Mumps Virus (n = 5)^d^
Demographics				
Gender (male), No. (%)	39 (59)	4 (57)	5/7 (71)	5 (100)
Age, y	35 (15–84)	45 (25–53)	32 (22–57)	39 (32–61)
Illness day on admission, d	5 (1–30)	5 (2–14)	3.5 (2–6)	3 (2–5)
Duration of hospital stay, d	5 (1–76)	5 (3–67)	2 (1–4)	4 (3–35)
HIV status, No. (%)				
Positive	0	0	0	0
Negative	24 (36)	1 (14)	4 (57)	1 (20)
Unknown	42 (64)	6 (86)	3 (43)	4 (80)
Clinical signs and symptoms, No. (%)				
Fever	58 (88)	7/7 (100)	6 /7 (86)	5 (100)
Headache	58 (88)	7/7 (100)	6 /7 (86)	5 (100)
Irritability	15 (23)	1/7 (14)	1/7 (14)	0
Lethargy	18 (28)	3/6 (50)	1/7 (14)	0
Vomiting	34 (52)	4/6 (67)	5/7 (71)	3 (60)
Seizures	23 (36)	2/6 (33)	0/7	2 (40)
Conscious	46 (70)	6/7 (86)	1/7 (14)	2 (40)
Skin rash	6 (9)	0/7	0/7	0
Hemiplegia	5 (8)	2/7 (29)	0/7	0
Paraplegia	1 (2)	0/7	1/7 (14)	0
Tetraplegia	1 (2)	0/6	0/7	0
Neck stiffness	45 (68)	6/7 (86)	5/7 (71)	3 (60)
Glasgow coma score of ≤8	7 (11)	3/7 (43)	0/7	1 (20)
Glasgow coma score of 9–12	16 (24)	2/7 (29)	1/7 (14)	1 (20)
Glasgow coma score of 13–15	43 (65)	2/7 (29)	6 /7 (86)	3 (60)
CSF cells and biochemistry				
White cells, cells/µL	101 (0–4183)	708 (38–1571)	503 (20–961)	683 (27–2146)
Neutrophils, No. (%)	13 (0–96)	9 (2–61)	24 (0–47)	18 (3–23)
Lymphocytes, No. (%)	86.5 (1–100)	91 (39–98)	76 (53–99.9)	82 (77–97)
Protein, g/L	0.7 (0.2–8.9)	1.36 (0.75–2.17)	0.71 (0.47–1.18)	0.67 (0.45–2.42)
CSF/blood glucose ratio	0.61 (0.34–1.04)	0.55 (0.47–0.61)	0.71 (0.59–0.85)	0.52 (0.49–0.81)
Lactate, mmol/L	2.65 (1.4–14.03)	3.52 (2.02–4.83)	2.5 (1.9–3.8)	2.9 (1.9–4.3)
Antiviral treatment, No. (%)				
Oral acyclovir	2 (3)	NA	NA	NA
Intravenous acyclovir	8 (13)	6/6 (100)	NA	NA
Oral valacyclovir	44 (72)	NA	NA	1 (20)
Modified Rankin Scale at discharge,^e^ No. (%)				
0	8 (13)	1/7 (14)	1/7 (14)	1 (20)
1	12 (19)	0	1/7 (14)	3 (60)
2	10 (15)	0	4/7 (58)	0
3	25 (39)	3/7 (43)	1/7 (14)	1 (20)
4	4 (6)	0	0	0
5	4 (6)	3/7 (43)	0	0
6	1 (2)	0	0	0

Continuous variables are presented as median (range).

^a^Denominators may vary slightly.

^b^Diagnosed by current standard tests for routine diagnosis.

^c^Diagnosed by mNGS, followed by PCR confirmatory testing.

^d^Diagnosed by current standard tests, expanded PCR testing, and mNGS combined.

^e^0: Full recovery with no symptoms; 1: No significant disability; 2: Slight disability; 3: Moderate disability; 4: Moderately severe disability; 5: Severe disability; and 6: Dead.

### An Overview of mNGS

**Figure 3.**
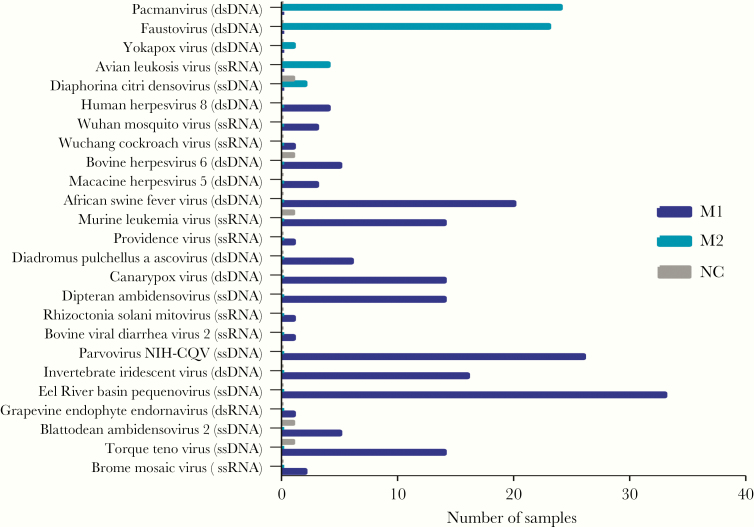
Bar chart showing the frequency of common contaminants and viruses of unknown neurotropic property (human herpes virus 8 and Torque teno virus) found in cerebrospinal fluid (CSF) samples by both DNA and RNA workflow and viruses in negative control CSF. Abbreviations: ds, double-stranded; ss, single-stranded.

The 68 included CSF samples (including 2 negative controls) were separately sequenced using both DNA and RNA virus workflows in a blinded fashion. Subsequently, a total of 62 565 802 and 49 233 869 reads were obtained from the DNA and RNA libraries, respectively (Supplementary Table 3). Sequences related to 29 viral species were detected, with 23 found in the RNA and 7 found in the DNA library (Figures 2 and 3). The detected viruses included viruses known to cause CNS infections and those with unknown neurotropic properties (Torque teno virus [n = 14] and herpes virus 8 [n = 4]). Additionally, previously reported common contaminants of the mNGS data set were also found [25, 26], almost exclusively in the RNA virus library (Figure 3).

### Detection of Viruses in CSF Samples That Were Positive by Routine Diagnosis

Of the 15 CSF samples positive either by PCR or serological testing as part of routine care, mNGS was able to detect a viral pathogen in 5/7 HSV-, 1/1 VZV-, 1/4 MuV-, 0/2 DENV-, and 0/1 JEV-positive samples (Figure 2). None of the HSV and VZV sequences were found in the library of the RNA virus workflow (Table 2).

**Table 2.** Results of Viral PCR and Metagenomic Analysis

CSF No.	Virus	Real-time PCR Ct Value	Detected by PCR as Part of Routine Care (Y/N)	Total Metagenomic Reads	No. of Unique Viral Reads	(%) of Viral Reads^a^	mNGS Library
1	HSV	25.01	Y	326 396	49	0.015	DNA
2	HSV	28.01	Y	588 504	184	0.031	DNA
3	HSV	30.36	Y	996 348	6	0.001	DNA
4	HSV	23.77	Y	1 145 710	243	0.021	DNA
5	HSV	28.71	Y	346 166	11	0.003	DNA
6	HSV	Unavailable	Y	1 345 954	0	0.000	NA
7	HSV	31	Y	891 566	0	0.000	NA
8	VZV	22.7	Y	1 335 288	152	0.011	DNA
9	Mumps	35.2	Y	975 714	6	0.001	RNA
10	Enterovirus	33.36	ND	539 752	21	0.004	RNA
11	Enterovirus	34.25	ND	635 310	38	0.006	RNA
12	Enterovirus	34.79	ND	765 564	10152	1.326	RNA
13	Enterovirus	34.78	ND	732 634	89	0.012	RNA
14	Enterovirus	31.23	ND	988 668	2415	0.244	RNA
15	Enterovirus	32.3	ND	594 964	100	0.017	RNA
16	Enterovirus	35.65	ND	543 912	21	0.004	RNA
17	Enterovirus	Negative	ND	579 486	2	0.000	RNA
18	Enterovirus	Negative	ND	571 902	2	0.000	RNA
19	Enterovirus	Negative	ND	720 042	4	0.001	RNA
20	Enterovirus	Negative	ND	511 608	1	0.000	RNA
21	Enterovirus	Negative	ND	818 654	2	0.000	RNA
22	Enterovirus	Negative	ND	513 428	5	0.001	RNA
23	Enterovirus	Negative	ND	1 197 290	13	0.001	RNA
24	Enterovirus	Negative	ND	923 908	4	0.000	RNA
25	Enterovirus	Negative	ND	993 918	1	0.000	RNA
26	Enterovirus	Negative	ND	1 302 784	20	0.002	RNA
27	Enterovirus	Negative	ND	1 628 722	7	0.000	RNA
28	Enterovirus	Negative	ND	1 181 716	24	0.002	RNA
29	Enterovirus	Negative	ND	926 462	22	0.002	RNA
30	Enterovirus	Negative	ND	938 524	20	0.002	RNA
31	Enterovirus	Negative	ND	1 028 194	12	0.001	RNA
32	Enterovirus	Negative	ND	1 239 458	4	0.000	RNA
33	Rotavirus	Negative	ND	1 176 486	24	0.002	RNA

^a^Denominators are the total reads of the corresponding samples.

Abbreviations: CSF, cerebrospinal fluid; mNGS, metagenomic next-generation sequencing; ND, not done; PCR, polymerase chain reaction.

Detection of sequences related to human pathogenic viruses in CSF that were negative by routine diagnosis, and results of PCR assessment of mNGS results

Of the 51 CSF samples that were negative by routine diagnosis, sequences related to neurotropic viruses were found in 24 (48%) samples by mNGS (Table 2). The detected viruses included enteroviruses (EVs; n = 23) and rotavirus (n = 1). Additionally, of the 2 CSF samples from non-CNS-affected patients, 1 had 4 sequences related to enterovirus detected by mNGS.

After PCR confirmation testing of CSF samples in which a viral hit was detected by mNGS, the rotavirus case and the negative control CSF, in which EV-related sequences were detected, became negative (Table 2). The number of EV-positive CSF samples was reduced from 23 to 7, with more enteroviral sequences being recorded in the PCR-confirmed group than in the unconfirmed group (Table 2). Of these, 3 had genome coverage of 61%, 78%, and 90%, including 1 echovirus 6 and 2 echovirus 30. Notably, the majority (12/16, 75%) of EV PCR-negative samples had EV reads identical to those obtained from samples with a high abundance of EV sequences (including samples #12 and #14), with which they shared an index (Supplementary Table 4), suggesting the potential of barcode bleedthrough during the sequencing procedure.

### Results of Expanded PCR Testing and Sensitivity Assessment of mNGS Using PCRs as Reference Assays

Because PCR testing for viruses (EVs and MuV) was not performed as part of routine diagnosis, to further assess the prevalence of these viruses in the study patients, we expanded PCR testing to CSF samples that were negative by mNGS analysis. Subsequently, only MuV was detected by PCR in 4 CSF samples, including 1 positive by both serological and mNGS methods (real-time PCR cycle threshold [Ct] values: 35), 2 positive by serological testing as part of standard care (Ct values: 36 and 40), and 1 negative by mNGS (Ct value: 40). Serological testing for MuV in this patient was not done as part of routine care (Supplementary Table 5). Thus a combination of serology and molecular assays (PCR and mNGS) increased the diagnostic yield from 22.7% (15/66) to 34.8% (23/66) (Figure 2).

mNGS identified a viral pathogen in 14/19 CSF samples that were positive by PCR analysis (including routine diagnosis and expanded testing). Additionally, mNGS detected reads related to EVs in 16/47 CSF samples that were negative by subsequent PCR analysis. Using PCRs as reference assays, the sensitivity and specificity of mNGS were 74% (14/19) and 66% (31/47), respectively. Of the PCR-positive samples, there was no difference in the leukocyte counts between the mNGS-negative and -positive groups (median [range], 331 [27–2146] vs 356 [22–4183]; *P = *.82).

### Rapid Detection of Encephalitis in CSF by MinION Nanopore Sequencing

**Figure 4.**
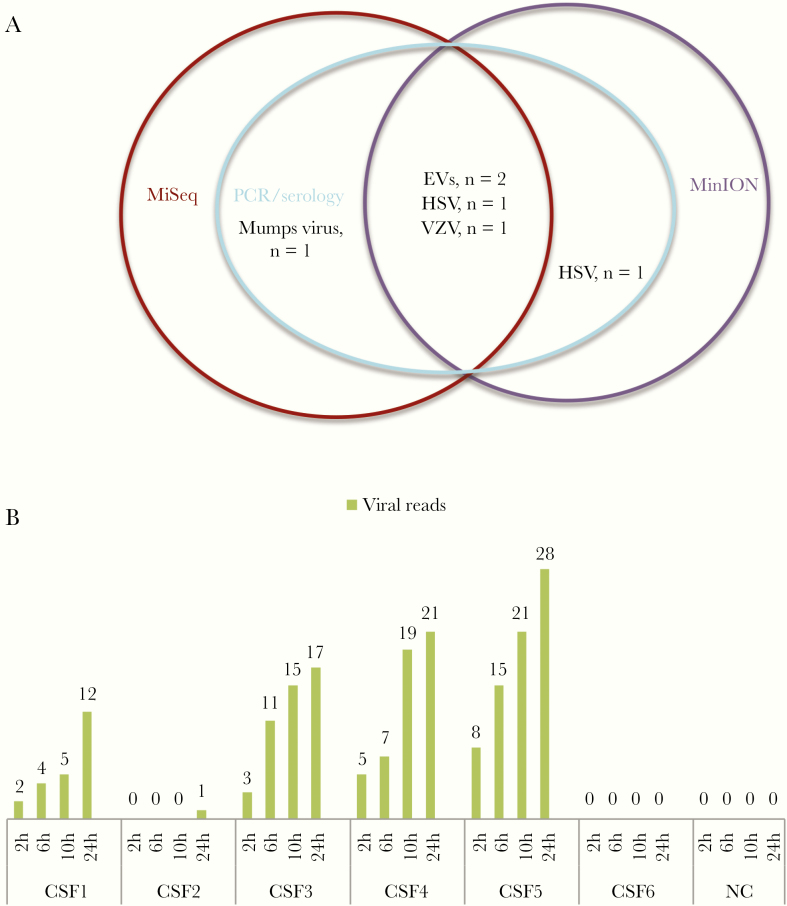
Results of MinION Nanopore–based metagenomics. A, Venn diagram showing the agreement between metagenomic approaches and polymerase chain reaction (PCR). B, Cumulative number of MinION reads assigned to corresponding viral species found in cerebrospinal fluid (CSF) by PCR at different time points. CSF1 and CSF2: herpes simplex virus (HSV) positive; CSF3: varicella zoster virus positive; CSF4 and CSF5: enterovirus positive; CSF6: mumps virus positive. All the 12 reads obtained from CSF1 were assigned to HSV1, and the single reads obtained from CSF2 were assigned to HSV2. Abbreviations: EV, enterovirus; HSV, herpes simplex virus; PCR, polymerase chain reaction; VZV, varicella zoster virus.

A MinION Nanopore–based metagenomic approach detected HSV (n = 2), VZV (n = 1), and EV (n = 2) in 5/6 CSF samples that were PCR positive for these viruses (Figure 4A). Of these 5 MinION-positive samples, 1 HSV sample was negative, and the other 4 were positive by MiSeq-based mNGS workflows (Figure 4A). Notably, after 2 hours of the sequencing run, reads assigned to corresponding viral species found in CSF by PCR were obtained in 4/5 MinION-positive samples. MinION, however, failed to detect MuV in a CSF sample that was positive by both PCR (Ct value = 36) and MiSeq workflow (Figure 4).

## Discussion

We report the results of an investigation assessing the utility of next/third-generation sequencing–based metagenomics as a hypothesis-free approach for detection of viral etiology in the CSF of 66 consecutively treated patients with meningoencephalitis. The patients were admitted to a tertiary referral hospital in Ho Chi Minh City, Vietnam, and the majority (51%) had moderate/severe disability at discharge. The results showed that in a single test metagenomics could accurately detect nucleic acids of a wide range of neurotropic viruses in the CSF of 66 participants, whose diagnoses were only established by extensive PCR testing targeted at a broad range of pathogens. Notably, of these 66 patients, 7 (11%) EV-infected patients were initially left undiagnosed at hospital discharge because physicians did not consider EV diagnosis as part of routine care. EV infection should therefore be considered as an important differential diagnosis in adults presenting with meningoencephalitis [27] and should be excluded (eg, by PCR testing) before mNGS analysis.

Although antivirals are currently not available for most encephalitis-causing viruses, rapid and accurate detection of viral etiology in patient samples remain essential to inform clinical management, such as avoiding unnecessary antibiotic prescription, and public health policy-makers. Thus, testing for a wide spectrum of pathogens is essential to maximize the diagnostic yield in patients presenting with meningoencephalitis. Under these circumstances, a single pan-pathogen assay such as mNGS is a useful approach, given the limited amount of CSF samples and resources available for microbial investigation, especially in low- and middle-income countries like Vietnam. However, the failure of mNGS to detect nucleic acids of JEV and DENV in serologically positive CSF samples emphasizes that testing for pathogen-specific antibodies remains an important diagnostic pathway in patients presenting with meningoencephalitis, as viral nucleic acids of some viruses (eg, flaviviruses) may not be present in the collected CSF.

The sensitivity of our mNGS workflows is comparable with that of recent mNGS studies [6, 9]. Low viral load may be a factor in the failure of mNGS to detect HSV and MuV in CSF samples with real-time PCR Ct values of 31 for HSV and 36, 40, and 40 for MuV. Because viral reads only accounted for a small proportion of total mNGS reads, increasing the sequencing depth per sample would likely increase the sensitivity of mNGS. However, this increases the sequencing costs.

Currently, there are no established robust criteria that can reliably define a true mNGS positive without the requirement of confirmatory testing. Criteria such as the presence of at least 3 reads mapped to 3 different genomic regions of a virus genome or the absence of viral reads in negative controls have recently been proposed [6, 10, 12]. Such approaches are hindered by the well-known cross-talk contamination phenomenon, occurring as part of the mNGS procedure [10], which, however, can be dramatically reduced through the use of the dual barcoding strategy, which was recently developed [28]. Because we did not employ the dual barcoding strategy, cross-talk contamination may explain the obtained specificity of 66%, which is lower than the reported data from a previous study [9]. Alternatively, the low specificity could be attributed to the degradation of stored viral RNA and/or the low abundance of viral RNA in the tested samples, leading to the failure of EV PCR to replicate some of the mNGS findings. Retrospectively, the specificity of mNGS would have increased to 83% if a threshold of ≥6 reads was considered positive (Table 2), suggesting a correlation between the number of mNGS reads and PCR confirmatory results. Collectively, the specificity of the mNGS-based diagnostic approach could potentially be improved through the use of a proper barcoding strategy and/or criteria such as those based on the number of unique viral reads obtained from a sample under investigation, which merits further research.

Recently, the single-molecule real-time sequencing developed by Oxford Nanopore Technologies has emerged as a promising tool for clinical settings because of its short turnaround time. As such, it could potentially overcome the current limitation of the long turnaround time posed by other NGS platforms. However, scarce information exists for the application of Oxford Nanopore Technologies as a hypothesis-free approach to detect viral agents in clinical samples [10, 29, 30]. The results of our complementary analysis demonstrate that MinION-based metagenomics could accurately detect viral pathogens in CSF samples within 2 hours after the sequencing run, whereas the current Illumina MiSeq–based metagenomic approach takes around 48–56 hours to complete. Collectively, the data suggest that the sensitivity of MinION is comparable with that of mNGS/PCR, and thus point to the utility potential of MinION sequencing for rapid diagnosis of meningoencephalitis, which merits further research.

Similar to previous reports [25, 26], numerous common contaminants of the mNGS data set (eg, parvovirus, densovirus) were found in both the DNA and RNA virus libraries in our study. Although it is likely that those contaminants were derived from laboratory reagents (eg, extraction kits) [25], their potential impacts on the performance of mNGS, especially in terms of sensitivity and specificity, remain unknown.

The strengths of our study include that it was conducted on consecutive cases, minimizing selection bias. CSF samples were analyzed individually, and mNGS hits were reconfirmed by specific PCR, allowing for back-to-back comparison between mNGS and viral PCR. However, our study has some limitations. First, it was conducted on stored CSF samples. Second, we only focused on viruses, while meningoencephalitis can be caused by nonviral agents such as intracellular bacteria (rickettsiae) [31]. Third, we did not test other clinical samples. Of note, JEV has recently been detected in the urine of patients presenting with meningoencephalitis [32, 33]. Last but not least, the inclusion of nontemplate controls in addition to the 2 noninfectious CSF samples would have better captured the spectrum of contaminations of the mNGS procedure.

To summarize, we report pioneering data on the performance of metagenomic next/third-generation sequencing on the CSF of meningoencephalitis patients in Vietnam, a resource-limited setting. The results shows that in a single assay, metagenomics was able to detect a wide spectrum of neurotropic viruses in CSF samples of meningoencephalitis patients, and thus it could potentially replace conventional nucleic acid–based diagnostic assays such as PCR. Further studies are needed to determine the clinical implications of real-time sequencing in the diagnosis and management of meningoencephalitis patients, especially in resource-limited settings, where pathogen-specific assays are limited in number.

## Supplementary Data

Supplementary materials are available at *Open Forum Infectious Diseases* online. Consisting of data provided by the authors to benefit the reader, the posted materials are not copyedited and are the sole responsibility of the authors, so questions or comments should be addressed to the corresponding author.

ofaa046_suppl_Supplementary_Table_2

ofaa046_suppl_Supplementary_Materials
