# Supplementary material for: Performance of Metagenomic Next-Generation Sequencing for the Diagnosis of Viral Meningoencephalitis in a Resource-Limited Setting
Source: Open Forum Infect Dis. 2020 Feb 8;7(3):ofaa046. doi: 10.1093/ofid/ofaa046 (PMC7051036; doi:10.1093/ofid/ofaa046)
Supplement: ofaa046_suppl_Supplementary_Materials [file ofaa046_suppl_supplementary_materials.docx]

**SUPPLEMENTARY MATERIALS FOR**

**Performance of metagenomic next-generation sequencing for the diagnosis of viral meningoencephalitis in a resource limited setting**

Nguyen Thi Thu Hong^1^, Nguyen To Anh^1^, Nguyen Thi Hoang Mai^1^, Ho Dang Trung Nghia^1,2^, Le Nguyen Truc Nhu^1^, Tran Tan Thanh^1^, Nguyen Hoan Phu^1,3^, Xutao Deng^4,5^, H. Rogier van Doorn^1,6^, Nguyen Van Vinh Chau^7^, Eric Delwart^4,5^, Guy Thwaites^1,6^ and Le Van Tan^1^

^1^Oxford University Clinical Research Unit, Ho Chi Minh City, Vietnam

^2^Pham Ngoc Thach University of Medicine, Ho Chi Minh City, Vietnam

^3^Department of Medicine, Vietnam National University, Ho Chi Minh City, Vietnam

^4^Vitalant Research Institute, San Francisco, California, USA

^5^Department of Laboratory Medicine, University of California, San Francisco, California, USA

^6^Centre for Tropical Medicine and Global Health, Nuffield Department of Medicine, University of Oxford, Oxford, UK

^7^Hospital for Tropical Diseases, Ho Chi Minh City, Vietnam

**Correspondence**:

Corresponding author: Le Van Tan, Oxford University Clinical Research Unit, Ho Chi Minh City, Vietnam; Tel: (+84 28) 8384009; Fax: (+84 8) 9238904; E-mail: [tanlv@oucru.org](mailto:tanlv@oucru.org)

Alternate corresponding author: Nguyen Thi Thu Hong, Oxford University Clinical Research Unit, Ho Chi Minh City, Vietnam; Tel: (+84 28) 8384009; Fax: (+84 8) 9238904; E-mail: [hongntt@oucru.org](mailto:hongntt@oucru.org)

**Word count:** abstract: 248 full text: 3513

**Keywords:** Metagenomics, Next-generation sequencing, Nanopore, MinION, meningoencephalitis

**Running title:** Metagenomic diagnosis of viral meningoencephalitis

**Supplementary Table 1.** Routine diagnostic workup during the study period at the study site

| **Suspected clinical entity** | **First line diagnosis** | **Additional testing*** |
| --- | --- | --- |
| Tuberculous meningitis | Ziehl Neelsen stain, GenXpert and culture^#^ | NA |
| Bacterial meningitis | Gram stain and culture |  |
| Meningoencephalitis | HSV PCR | VZV PCR and serological testing for flaviviruses (including JEV and DENV), and mumps virus |
| Cryptococcus | Lateral Flow Assay | NA |
| Auto-immune encephalitis | Not done | Anti-NMDAR encephalitis** |

**Note to Table S1.** *Up on requested by treating physicians, ^#^ using Mycobacteria Growth Indicator tubes, **retrospective testing

**Supplementary Table 2:** Results of routine diagnosis, expanded PCR testing and mNGS analysis

**Supplementary Table 3**: mNGS reads obtained from DNA/RNA-virus workflows

|  | Total reads | Median | Range |
| --- | --- | --- | --- |
| DNA-virus workflow | 62,565,802 | 859,656 | 1,487,000 – 2,125,00 |
| RNA-virus workflow | 49,233,869 | 717,707 | 7,368 – 5,874,00 |

**Supplementary Table 4**: EV reads of PCR negative samples identical to reads found in sample(s) with a high abundance of EV reads with which they (did not) share(d) an index

| **CSF number** | **Number of unique EV reads found in PCR negative samples** | **Number of unique EV reads (%) (1)** | **Number of unique EV reads (%) (2)** |
| --- | --- | --- | --- |
| 17 | 2 | 1 (50) | 0 |
| 18 | 2 | 2 (100) | 0 |
| 19 | 4 | 0 | 0 |
| 20 | 1 | 0 | 0 |
| 21 | 2 | 2 (100) | 0 |
| 22 | 5 | 5 (100) | 0 |
| 23 | 13 | 4 (31) | 0 |
| 24 | 4 | 2 (50) | 0 |
| 25 | 1 | 0 | 0 |
| 26 | 20 | 5 (25%) | 0 |
| 27 | 7 | 4 (57) | 0 |
| 28 | 24 | 12 (50) | 0 |
| 29 | 22 | 0 | 0 |
| 30 | 20 | 2 (10) | 0 |
| 31 | 12 | 7 (58) | 0 |
| 32 | 4 | 2 (50) | 0 |

^\^

**Note to Supplementary Table 4:**

(1) identical to reads found in sample(s) with a high abundance of EV reads with which it shared an index.

(2) identical to reads found in sample(s) with a high abundance of EV reads with which it did shared an index.

**Supplementary Table 5:** Result summary of mumps virus diagnostics

| **Patient ID** | **Serology** | **RT-PCR** | **mNGS** |
| --- | --- | --- | --- |
| 1 | Positive | Negative | Negative |
| 2 | Positive | Positive | Positive |
| 3 | Positive | Positive | Negative |
| 4 | Positive | Positive | Negative |
| 5 | Not done | Positive | Negative |
